# Electrocatalytic Activity of Electrospun Multi-Walled Carbon Nanotubes/Poly(3-aminobenzylamine) Composite for Detection of Dopamine in Human Urine

**DOI:** 10.3390/bios16040226

**Published:** 2026-04-20

**Authors:** Tharathip Khueanpech, Saengrawee Sriwichai

**Affiliations:** 1 Department of Chemistry, Faculty of Science, Chiang Mai University, Chiang Mai 50200, Thailand; tharathip_k@cmu.ac.th; 2 Center of Excellence for Innovation in Chemistry (PERCH-CIC), Faculty of Science, Chiang Mai University, Chiang Mai 50200, Thailand

**Keywords:** conducting polymer, poly(3-aminobenzylamine), multi-walled carbon nanotube, dopamine, electrochemical

## Abstract

A nanostructured sensing platform based on electrospun functionalized multi-walled carbon nanotubes/poly(3-aminobenzylamine) (FMWCNTs/P3ABA) was developed for the electrochemical detection of dopamine (DA) on fluorine-doped tin oxide (FTO) glass substrate. The electrochemical characteristics of the electrodes were investigated by chronocoulometry (CC) and cyclic voltammetry (CV) in phosphate-buffered saline solution containing K_3_[Fe(CN)_6_] as a redox mediator. The zeta potential analysis confirmed the presence of a stable surface charge that favors electrostatic interaction with DA molecules. The DA detection was performed in human urine by differential pulse voltammetry (DPV) over a potential of −0.2 to 0.8 V and at scan rate of 5 mV s^−1^, where the FMWCNTs/P3ABA nanofiber electrode exhibited a high sensitivity of 1.502 µA cm^−2^ nM^−1^, a linear detection range of 10–500 nM (R^2^ = 0.992), and a limit of detection of 1.753 nM. The sensor exhibited stable and reproducible responses, and the fibrous composite effectively discriminated DA from common electroactive interferents, including ascorbic acid, uric acid, creatinine, and glucose. Furthermore, reliable dopamine quantification in human urine samples demonstrates the strong potential of the electrospun FMWCNTs/P3ABA composite nanofiber platform for practical bioanalytical and non-invasive sensing applications in the future.

## 1. Introduction

Electrically conductive polymers have been recognized as attractive materials for chemical and biological sensing due to their tunable redox properties, mechanical flexibility, and ease of chemical modification. Polymers derived from aniline, pyrrole, acetylene, and thiophene provide versatile redox-active frameworks that can be adapted for detecting a wide range of molecular targets [1,2,3]. Among these, poly(3-aminobenzylamine) (P3ABA), a structurally modified form of polyaniline, has emerged as a particularly strong candidate. Its amine-rich backbone could promote molecular accumulation and selectivity through hydrogen bonding and electrostatic interactions with dopamine molecules. These characteristics have enabled P3ABA and related polymers to serve effectively in platforms designed for hormone and neurotransmitter monitoring, including dopamine (DA), cortisol, and other small biomolecules [4,5]. Recent advancements in polymer-based electrochemical sensors have increasingly focused on composite materials. Incorporating conducting polymers with nanoscale carbon structures, metallic nanoparticles, or metal oxides has demonstrated substantial improvements in signal response, stability, and selectivity [6]. For instance, hybrid microfiber mats fabricated from PLA/PBAT and graphite through electrospinning have shown high responsiveness toward DA, whereas MnO_2_ and AuNP-modified PANI electrodes are suitable for glucose sensing without requiring enzymatic reagents [7]. Polypyrrole-molybdenum oxide bilayers coatings on ITO substrates have also enabled selective DA oxidation even in the presence of biologically abundant interferents [8]. These studies collectively highlight the benefits of integrating nanomaterials into polymer matrices to enhance electrochemical performance. Currently, the incorporation of conducting polymers and/or their derivatives with nanomaterials such as carbon nanotubes (CNTs) [9,10,11,12] and gold (Au) [7]. These are good materials for developing electrochemical biosensors.

Carbon nanotubes particularly multi-walled CNTs (MWCNTs) functionalized with carboxyl groups, have attracted significant attention due to their ability to facilitate charge transport and provide a highly accessible surface area [13,14]. Functionalization not only improves their dispersibility in polymer solutions but also allows them to form strong interfacial interactions with polymer chains, resulting in composites with enhanced electroactive surface area and improved catalytic behavior [10]. Such advantages make functionalized MWCNTs ideal for biosensing interfaces that require high sensitivity and stability under complex chemical environments. In addition, these properties play a crucial role in enhancing sensor performance for dopamine detection. The increased electroactive surface area provides more active sites for the oxidation of DA, leading to increased current responses. Furthermore, the improved electron transfer kinetics facilitate faster and efficient redox reactions of DA at the electrode surface [11].

Dopamine (DA), a critical neurotransmitter involved in motor control, cognition, and neuroendocrine regulation, must be tightly regulated within the human body. Abnormal DA concentrations are associated with several neurological disorders, including Parkinson’s disease, schizophrenia, and depressive disorders [15,16]. In physiological fluids, DA typically exists at low micromolar to nanomolar levels and is accompanied by structurally similar electroactive species such as uric acid (UA) and ascorbic acid (AA), both of which exhibit overlapping oxidation potentials on most electrode surfaces. This creates a fundamental challenge for achieving selective detection in real biological matrices [17]. In fact, the normal DA concentration in urine for a healthy person is extremely low (274 to 500 nM) [18]. A variety of sensing platforms have been introduced to address this issue including COF-modified carbon fiber electrodes for real-time monitoring of DA in disease-model brain tissues [19], microneedle sensors based on molecular imprinting for label-free neurotransmitter recognition [20], and graphene-derived nanocomposites capable of simultaneous DA and UA measurement [21]. The incorporation of graphene oxide (GO), SiO_2_, and PANI has also shown promise for DA quantification in urine at concentrations relevant to point-of-care diagnostics [22]. Across these studies, electrochemical methods especially cyclic voltammetry (CV), differential pulse voltammetry (DPV), amperometry, and impedance spectroscopy (EIS) have been extensively employed because they offer rapid analysis, portability, and low operational cost [22,23,24]. Despite the progress made, achieving a sensing surface that is both highly selective and stable in the presence of biologically common interferents continues to be an important objective [25,26]. Tailoring electrode surfaces through electrospinning has therefore become increasingly appealing. The technique enables the formation of continuous nanofiber networks with large surface-area-to-volume ratios, interconnected porous structures, and enhanced accessibility for analyte transport [27,28]. Electrospun materials have been explored widely in tissue engineering, pharmaceutical delivery systems, and catalytic supports, and their structural attributes translate effectively to biosensing applications by supporting efficient electron transfer and molecular adsorption [29,30,31].

In this present study, an electrochemical dopamine sensor was fabricated using electrospun composite fiber of functionalized multi-wall carbon nanotubes (FMWCNTs) and P3ABA. The fabrication approach involved generating nanofibrous films directly on fluorine-doped tin oxide (FTO) substrates, enabling the formation of a conductive and highly porous active layer. Electrochemical assessments using chronocoulometry (CC), cyclic voltammetry (CV) and differential pulse voltammetry (DPV) demonstrated that incorporating FMWCNTs substantially strengthened the sensitivity, selectivity, and operational consistency of the sensor for detection of DA. The developed DA sensor also demonstrated reliable detection performance in human urine, supporting its potential use in real biological samples. In addition, the attenuated total reflectance Fourier-transform infrared spectroscopy (ATR-FTIR), scanning electron microscopy (SEM), transmission electron microscopy (TEM), X-ray photoelectron spectroscopy (XPS), Brunauer–Emmett–Teller (BET) surface area analysis and zeta potential measurements of the electrospun nanofiber films were also performed.

## 2. Experimental Section

### 2.1. Chemicals and Reagents

3-Aminobenzylamine (ABA), dopamine (DA), glucose (Glu), lactic acid (LA), creatinine (Cr), polyacrylonitrile (PAN), dimethylformamide (DMF), potassium chloride (KCl), phosphate-buffered saline (PBS) tablets, and fluorine-doped tin oxide (FTO) glass substrates were obtained from Sigma-Aldrich (Darmstadt, Germany). Ascorbic acid (AA) and uric acid (UA) were obtained from Poch (Gliwice, Poland) and Bio Basic (Markham, ON, Canada), respectively. Potassium hexacyanoferrate(III) (K_3_[Fe(CN)_6_]) and ammonium persulfate (APS) were purchased from Scharlau (Barcelona, Spain) and RCI Labscan (Bangkok, Thailand), respectively. Mass spect gold human urine, catecholamine free were acquired from Goldenwest diacnotics, LLC (Temecula, CA, USA). All chemicals were used as received without further treatment. Deionized water was used throughout all preparations. P3ABA was synthesized following the established procedure described in our earlier work [10,32]. FMWCNTs were produced via oxidative acid treatment of commercial MWCNTs (Nanogen, Chiang Mai, Thailand) using a nitric acid and sulfuric acid (RCI Labscan, Bangkok, Thailand) mixture (1:3 *v*/*v*), yielding nanotubes enriched with carboxyl functionalities [10,33].

### 2.2. Instrumentation

The electrospun fibers were examined using X-ray photoelectron spectroscopy (XPS; AXIS Ultra DLD, Manchester, UK) and attenuated total reflectance Fourier-transform infrared spectroscopy (ATR-FTIR; Bruker Tensor 27, Billerica, MA, USA). The morphological characteristics were obtained using field emission scanning electron microscopy (FE-SEM; JEOL JSM-6335F, Tokyo, Japan) and transmission electron microscopy (TEM; JEOL JEM-2010, Tokyo, Japan). The specific surface area and pore size distribution were determined using surface area and pore size analyzer (BET; QUANTACHROME NOVA2200E, Boynton Beach, FL, USA). The surface zeta potential was measured using Nano particle size, zeta potential (HORIBA SZ-100-S, Tokyo, Japan). Electrochemical measurements including chronocoulometry (CC), cyclic voltammetry (CV) and differential pulse voltammetry (DPV) were conducted with a PalmSens4 potentiostat (PalmSens, Houten, The Netherlands). A standard three-electrode configuration was used with platinum wire as the counter electrode, Ag/AgCl (3 M KCl) as the reference electrode, and a FTO substrate coated with the electrospun film as the working electrode.

### 2.3. Preparation of Modified Electrodes

The mixture of P3ABA (3% *w*/*v*), FMWCNTs (0.05% *w*/*v*), and PAN (5% *w*/*v*) in DMF was sonicated for 1 h and centrifuged at 4000 rpm for an additional hour to produce a uniform solution for the electrospinning process. Electrospinning was carried out directly onto the FTO substrate under optimized parameters, i.e., 15 kV potential, 1.0 mL h^−1^ flow rate, 10 cm tip-to-collector distance, and 10 min deposition time [10]. The resulting nanofiber mats were dried at room temperature and used as working electrode in subsequent electrochemical tests. The geometrical surface area of the working electrode is 1 cm^2^.

### 2.4. Electrochemical Measurement

Before dopamine sensing experiments, the redox behavior of the FMWCNTs/P3ABA-coated FTO electrode was assessed in PBS containing 0.5 mM K_3_[Fe(CN)_6_] and 0.1 M KCl using a CC measurement with a double potential step to estimate the electroactive surface area (EASA). In addition, the quasi-reversible diffusion control process of the electrodes was studied using CV. The electrochemical performance of the electrode was evaluated for DA sensing using DPV in PBS containing 0.5 mM K_3_[Fe(CN)_6_] and 0.1 M KCl at a scan rate of 5 mV s^−1^ over a potential from −0.2 to 0.8 V. The selectivity toward DA was investigated by introducing 1 mM of potential interferents, including ascorbic acid (AA), uric acid (UA), creatinine (Crn), and glucose (Glu). Reproducibility was assessed using three independently prepared electrodes in the presence of 1 mM DA, while repeatability was evaluated through ten consecutive measurements using a single electrode. Long-term stability was examined by storing the electrodes under ambient conditions and periodically recording their electrochemical responses over 30 days. For sensitivity study, the DPV calibration curves were constructed by successive additions of DA (10–1000 nM) into human urine sample.

### 2.5. Real Sample Analysis

To evaluate the practical applicability of the sensor, DA was spiked into human urine (5% *v*/*v* in PBS containing 0.5 mM K_3_[Fe(CN)_6_] and 0.1 M KCl) at concentrations ranging from 10 to 1000 nM. The DPV measurements were performed using three independently prepared P3ABA and FMWCNTs/P3ABA nanofiber electrodes within a potential of −0.2 to 0.8 V at a scan rate of 5 mV s^−1^. The calibration plots were constructed based on the corresponding peak currents. The recovery values were calculated to assess the analytical accuracy in the complex urine matrix. The sensor sensitivity was determined from the slope of the calibration curve obtained from the DPV responses, reflecting the electrode’s ability to detect small variations in DA concentration in real sample conditions.

## 3. Results and Discussion

### 3.1. Characterization of FMWCNTs/P3ABA Electrospun Nanofiber Film

**Figure 1:**
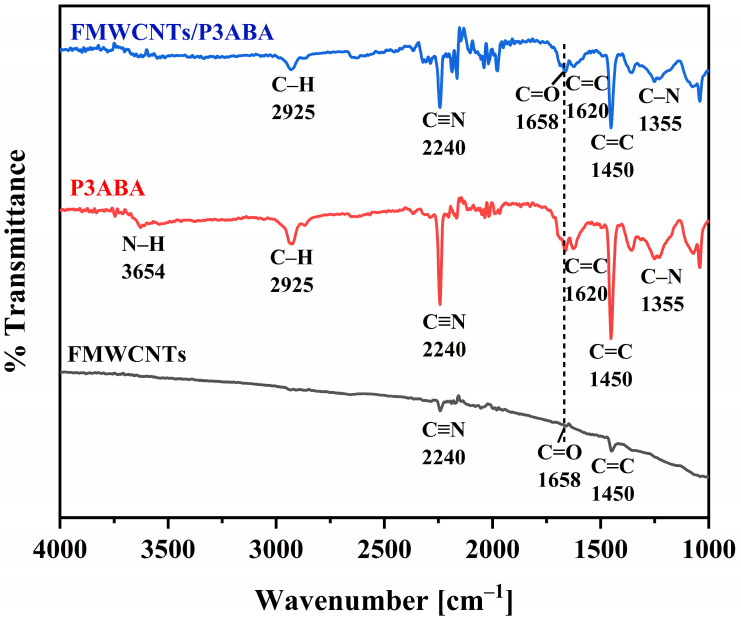
ATR-FTIR spectra of FMWCNTs, P3ABA, and FMWCNTs/P3ABA composite films.

The electrospun films were fabricated on a FTO-coated glass substrate with the optimized electrospinning condition [10]. The chemical compositions were studied using ATR-FTIR and XPS. The morphological characteristics were studied by SEM and TEM. The textural and surface charge properties were further investigated by BET surface area analysis and zeta potential measurements, respectively. The functional groups and chemical composition of the electrospun nanofiber films were investigated using ATR-FTIR spectroscopy, as shown in Figure 1. The peak at 2240 cm^−1^ corresponds to the stretching vibration of the C≡N bond originating from PAN residues which was employed as blending agent for electrospinning process [34,35]. For FMWCNTs, the absorption peak at 1658 cm^−1^ is attributed to the stretching vibration of the C=O bond of carboxylic groups introduced during the functionalization process of MWCNTs, whereas the band at 1450 cm^−1^ is associated with the stretching vibration of the C=C bond in the aromatic ring structure [36]. For P3ABA and FMWCNTs/P3ABA composite nanofibers, the similar spectral features were observed, indicating that the incorporation of FMWCNTs did not significantly alter the chemical structure of the polymer matrix. The absorption band at 3654 cm^−1^ is assigned to the N–H stretching vibration of the amine group, while the peak at 2925 cm^−1^ corresponds to the C–H stretching vibration of the aromatic ring in the P3ABA backbone [10]. In addition, the stretching vibrations of the C=C bond of the benzenoid rings and the C–N bond of the aromatic amine group of P3ABA appear at 1450 and 1355 cm^−1^, respectively [10,37]. The presence of FMWCNTs in the FMWCNTs/P3ABA composite film could not be clearly distinguished by the FTIR spectroscopy due to the overlap of characteristic absorption bands and the relatively low loading of FMWCNTs. Therefore, other surface-sensitive characterization techniques, i.e., XPS, were further employed to confirm the incorporation of FMWCNTs into the FMWCNTs/P3ABA composite nanofiber film [38].

**Figure 2:**
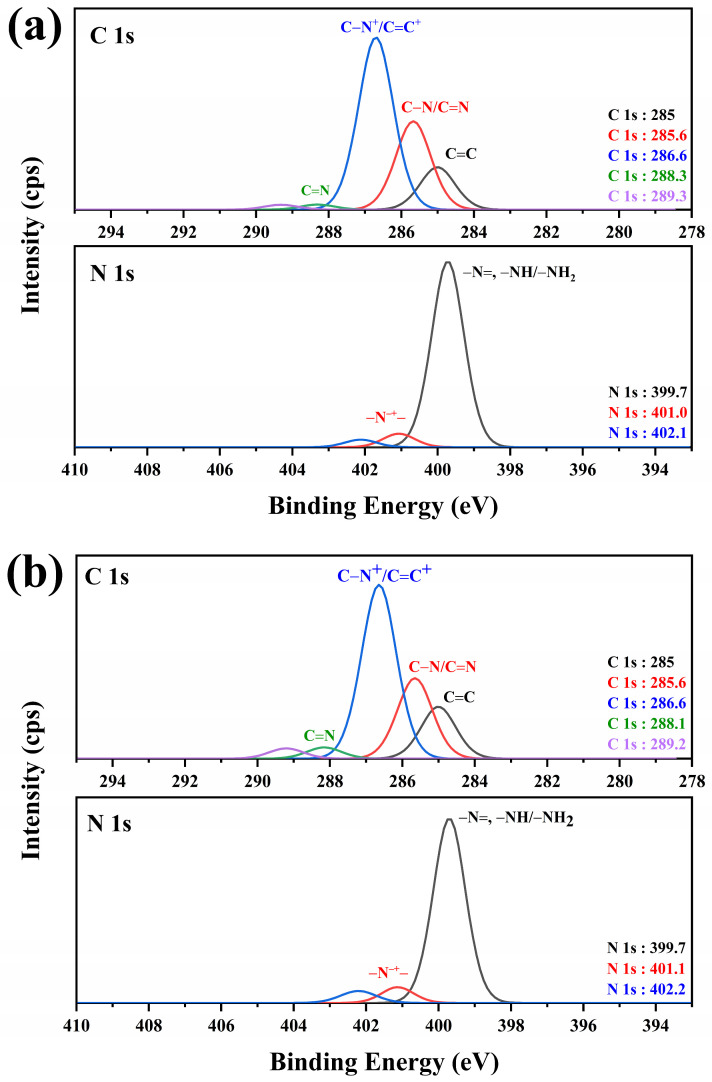
XPS spectra of (**a**) P3ABA and (**b**) FMWCNTs/P3ABA electrospun nanofiber films.

XPS was employed to further elucidate the chemical composition, bonding states, and interfacial interactions of the electrospun nanofiber films, as shown in Figure 2. The high-resolution C1s spectra of P3ABA and FMWCNTs/P3ABA, as shown in Figure 2a,b, appeared at 285, 285.6, 286.6, and 288.3 eV, which are attributed to C=C, C–N/C=N, C–N+/C=N+, and C=N corresponding to the benzenoid and quinoid structures in the P3ABA chain [39,40,41,42]. The ratio of the C=N (quinoid, 398.5–399.0 eV) and C-NH (benzenoid, 399.7–400.5 eV) component was increased from 1.9 (for P3ABA) to 2.1 (FMWCNTs/P3ABA), which indicating the enhanced oxidation of the polymer chains and suggesting strong π–π interactions and charge transfer between P3ABA and the FMWCNTs [43,44]. In addition, the N1s spectra show two characteristic components at approximately 399.7 eV and 401.0–401.1 eV, assigned to neutral amine/imine species (–N=, –NH, –NH_2_) and positively charged –N•^+^– species, respectively [45]. Notably, the ratio of neutral amine/imine and –N•^+^– was changed from 0.10 for P3ABA film to 0.11 for FMWCNTs/P3ABA composite films, the proportion of –N•^+^– was observed to be higher than P3ABA, which could be indicated for electronic interactions and partial charge transfer between P3ABA and FWCNTs in the composite film [46]. The XPS results could be summarized to state that the FMWCNTs/P3ABA composite film was successful in this study.

**Figure 3:**
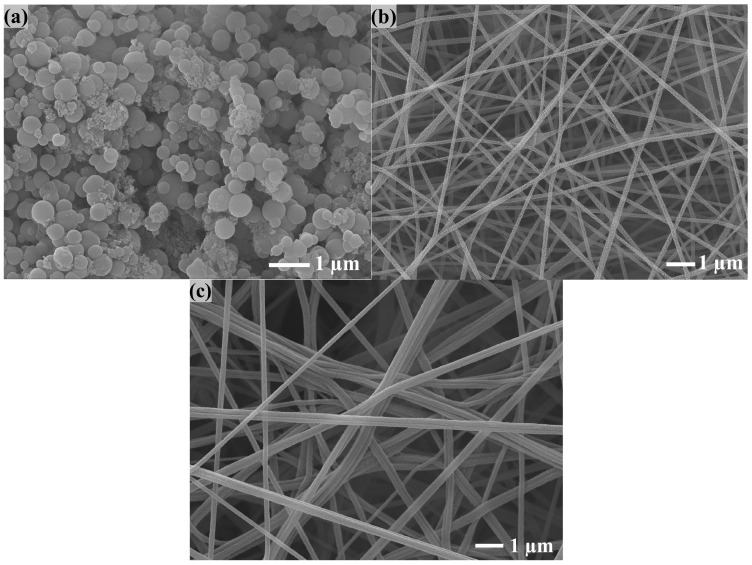
SEM images of (**a**) P3ABA powder, (**b**) P3ABA and (**c**) FMWCNTs/P3ABA electrospun nanofiber films.

**Figure 4:**
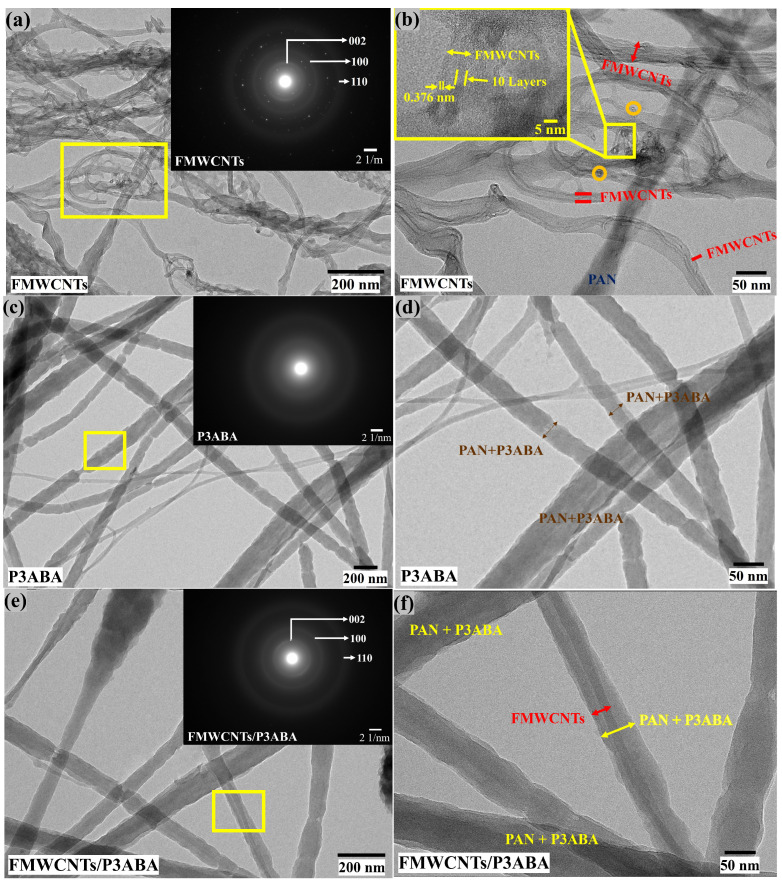
TEM images (**left**) of electrospun nanofiber films with SAED patterns and HR-TEM images (**right**) of (**a**,**b**) FMWCNTs, (**c**,**d**) P3ABA, and (**e**,**f**) FMWCNTs/P3ABA.

To investigate the morphology of the electrospun nanofiber film, the top view of the films was performed using SEM in Figure 3. It can be observed that the surfaces of the electrospun nanofibers were uniform and bead-free as seen for P3ABA powder (Figure 3a), P3ABA electrospun film (Figure 3b) and FMWCNTs/P3ABA electrospun film (Figure 3c). The average diameter of electrospun nanofibers were 134.76 ± 16.66 and 157.08 ± 17.96 nm, respectively. The composite fiber sizes increased, which could confirm the incorporation of FMWCNTs in P3ABA structures [10]. The TEM images provided the microstructural configuration of FMWCNTs, P3ABA, and FMWCNTs/P3ABA nanofibers as shown in Figure 4. As depicted in Figure 4a,b, the FMWCNTs exhibited a typical multi-walled tubular structure with clear graphitic layers, while the selected area electron diffraction (SAED) pattern (inset) revealed distinct diffraction rings indexed to the (002), (100), and (110) planes, confirming their crystalline nature [47,48]. The high-resolution TEM image further indicated an interlayer spacing of approximately 0.376 nm, with 10–15 nm diameter [49,50]. Figure 4c,d show the TEM images with halo SAED pattern of the electrospun P3ABA nanofibers, which display smooth, continuous, and uniform fibrous morphologies with an amorphous diffraction pattern, indicating the polymeric nature of P3ABA [51]. In contrast, the TEM images of FMWCNTs/P3ABA nanofibers as shown in Figure 4e,f clearly demonstrated the successful incorporation and homogeneous distribution of FMWCNTs within the P3ABA matrix. The FMWCNTs are well embedded along the fiber axis without noticeable aggregation, suggesting strong interfacial interactions between P3ABA and FMWCNTs [44,45]. Consequently, the TEM results could confirm the successful formation of FMWCNTs/P3ABA composite nanofiber with a well-integrated hybrid structure.

**Figure 5:**
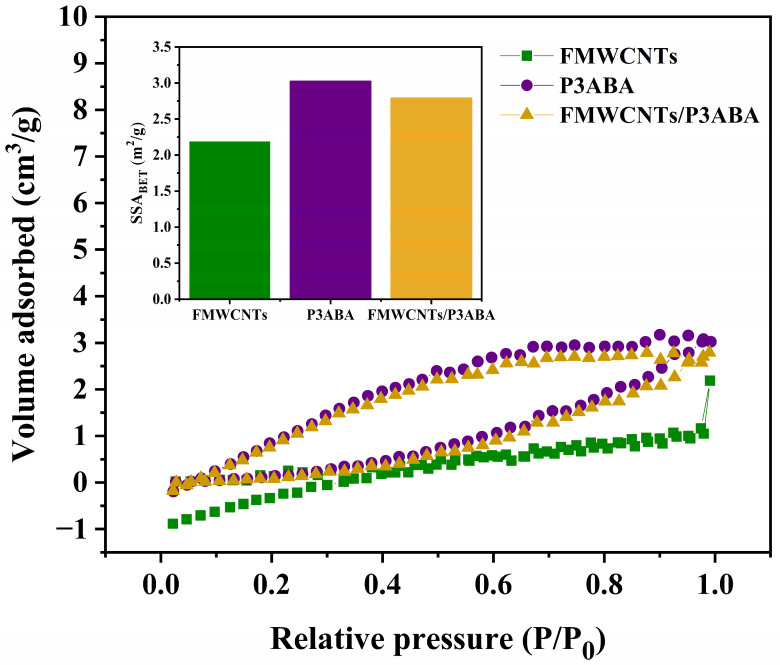
N_2_ adsorption–desorption isotherms and the specific surface area (SSA_BET_, inset) of FMWCNTs, P3ABA, and FMWCNTs/P3ABA.

Figure 5 shows the nitrogen adsorption–desorption isotherms and the corresponding BET specific surface areas of FMWCNTs, P3ABA, and FMWCNTs/P3ABA nanofibers. All samples exhibit nitrogen sorption isotherms that can be classified as IUPAC type III, indicating unbarred multilayer adsorption, which are typically associated with weak adsorbate–adsorbent interactions and nonporous or loosely aggregated materials with mesopore-like voids originating from interparticle or interfiber spaces rather than intrinsic porosity [52]. The gradual increase in adsorbed nitrogen over the entire relative pressure range (P/P_0_ = 0–1.0) further supports the absence of well-defined microporous structures. Among the investigated samples, P3ABA shows the highest nitrogen uptake across the relative pressure range, followed by the FMWCNTs/P3ABA composite, while FMWCNTs exhibit the lowest adsorption capacity. The BET specific surface areas (inset of Figure 5) are approximately 2.2, 3.0, and 2.8 m^2^ g^−1^ for FMWCNTs, P3ABA, and FMWCNTs/P3ABA, respectively. The slight decrease in surface area after incorporating FMWCNTs into the P3ABA matrix may be attributed to partial coverage of the polymer surface and the embedding or aggregation of nanotubes within the electrospun nanofiber network, which can reduce the number of accessible adsorption sites [53]. Nevertheless, the FMWCNTs/P3ABA composite still exhibits a higher surface area than pristine FMWCNTs, indicating that the electrospun nanofibrous structure effectively preserves accessible surface area. Moreover, the presence of mesopore-like voids between fibers and nanoparticles is expected to facilitate electrolyte diffusion and mass transport, which is advantageous for electrochemical applications such as sensing and charge-transfer processes [54,55].

The zeta potential values of FMWCNTs, P3ABA, and FMWCNTs/P3ABA nanofiber films in the absence and presence of 20 nM DA were investigated in the PBS containing 0.5 mM K_3_[Fe(CN)_6_] and 0.1 M KCl, as summarized in Table 1. In the absence of DA, the FMWCNTs exhibit a slightly negative zeta potential (−2.4 mV), which can be attributed to the presence of oxygen-containing functional groups on the nanotube surface generated during functionalization. In contrast, the P3ABA shows a positive zeta potential (9.3 mV), reflecting the protonation of amine and imine groups along the conducting polymer backbone under the measurement conditions [56]. Notably, the FMWCNTs/P3ABA composite displays a markedly negative zeta potential (−45.7 mV), indicating that the surface charge is dominated by the negatively charged FMWCNTs and suggesting strong interfacial interactions within the composite structure [57]. Upon the addition of 20 nM DA, significant changes in the zeta potential are observed. The zeta potential of P3ABA increases substantially to 25.6 mV, implying enhanced electrostatic interactions and adsorption of DA molecules on the positively charged polymer surface [58]. Meanwhile, the zeta potential values of FMWCNTs and FMWCNTs/P3ABA shift toward near-neutral values (−1.2 and −1.4 mV, respectively), indicating partial charge screening and competitive adsorption between DA and ferri/ferrocyanide redox species at the interface [59]. These results demonstrate that DA strongly influences the interfacial charge environment, which is closely related to the improved electrochemical response of the FMWCNTs/P3ABA composite toward dopamine sensing.

### 3.2. Electrochemical Characterization of the Electrospun Nanofiber Films

**Figure 6:**
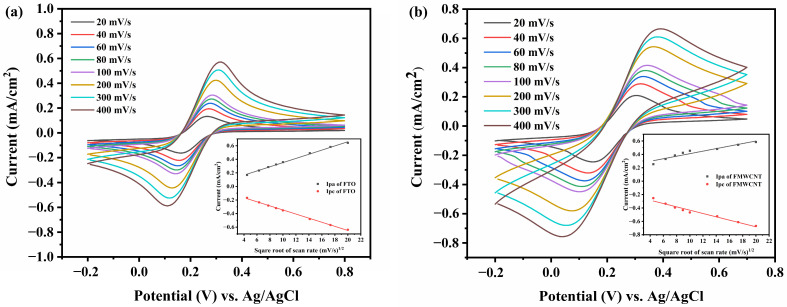

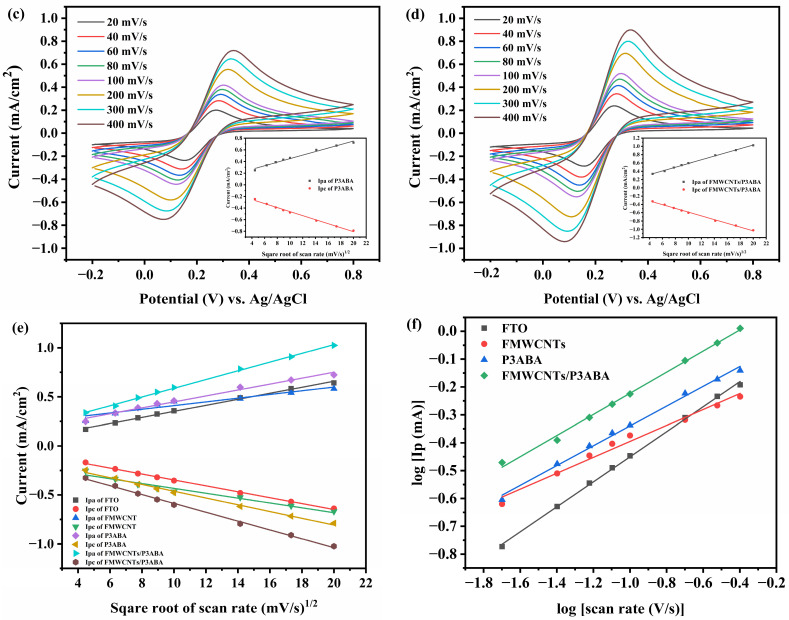
Cyclic voltammograms of (**a**) FTO, (**b**) FMWCNTs (**c**) P3ABA, and (**d**) FMWCNTs/P3ABA electrospun nanofiber films at different scan rates in a PBS solution containing 0.5 mM K_3_Fe(CN)_6_ and 0.1 M KCl with (**e**) linear responses of the peak currents and square root of the scan rate and (**f**) logarithm plot of the peak current (log Ipa) versus logarithm of the scan rate (log v).

The electroactivity of electrospun nanofiber P3ABA and FMWCNTs/P3ABA composite film was investigated as shown in Figure 6. The electrospun nanofiber films were studied in a PBS solution (pH 7) containing 5 mM K_3_Fe(CN)_6_ and 0.1 M KCl at various scan rates (20–400 mV/s) in the potential range of −0.2 to 0.8 V using CV technique prior to use as an electrochemical dopamine sensor. The CV responses of the fabricated electrospun nanofiber films were observed that the anodic peak to the cathodic peak clearly separated, which at different scan rates as shown in Figure 6a–d. According to the Randles-sevcik equation, the linear relationship of the peak currents (anodic peak current, Ipa and cathodic peak current, Ipc) versus square root of the scan rates (v^1/2^), as shown in Figure 6e, indicated the quasi-reversible diffusion control at the electrode surface [60]. The average difference between anodic and cathodic peak potentials (ΔEa,c) were 0.373, 0.246, 0.186, and 0.179 V for FTO, FMWCNTs, P3ABA, and FMWCNTs/P3ABA, respectively. The FMWCNTs/P3ABA composite presented the lowest values of ΔEa,c which could imply to the electrochemical enhancement of FMWCNTs/P3ABA composite. Furthermore, the slope of the log–log plot of Ipa vs. scan rate (v), as shown in Figure 6f, could theoretically predict the diffusion and adsorption controlled processes [61]. The slopes of the FTO, FMWCNTs, P3ABA and FMWCNTs/P3ABA electrodes were 0.449, 0.286, 0.355, and 0.379, respectively, which indicates the quasi-reversible diffusion process.

**Figure 7:**
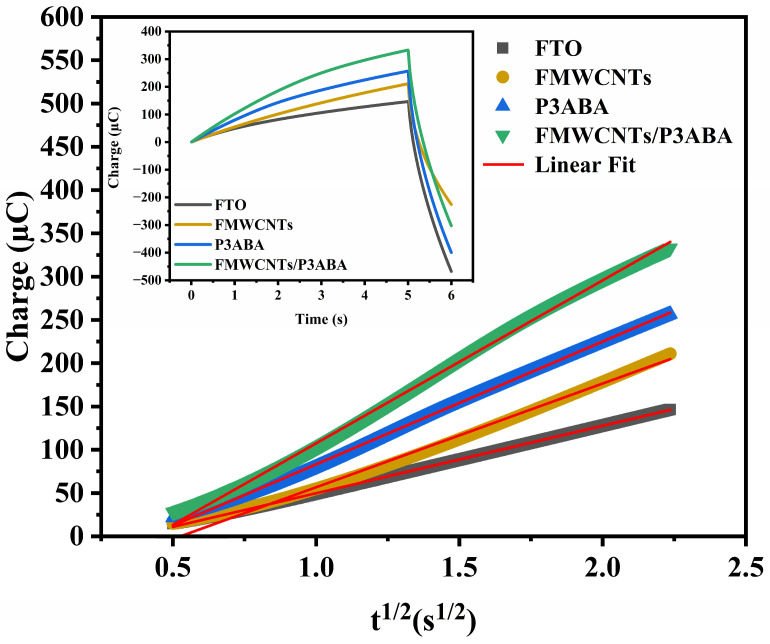
Anson plots by chronocoulometry (*Q* vs. *t*^1/2^) and inset is a comparative (*Q* vs. *t*), double potential step of FTO, FMWCNTs, P3ABA and FMWCNTs/P3ABA nanofiber films.

To characterize the electrochemical properties of the electrospun nanofiber films, the electroactive surface areas (EASAs) were calculated using the Cottrell equation [Equation (1)] [62]:

(1) $$Q\left(t\right)=\frac{2nFAD^{1/2}C_{R}t^{1/2}}{\pi^{1/2}}+c$$

where *n* is the number of the electrons transferred during the electrode reaction, *F* is the Faraday constant (96,485 C/mol), *A* is the effective surface area, *D* refers to the diffusion coefficient of K_3_[Fe(CN)_6_] in 0.1 M KCl (6.97 × 10^−6^ cm^2^/s), *C_R_* is the interfacial concentration of the redox probe (R), *t* is time, and *c* refers to the constant of integration and it can be related to minor contributions from double-layer charge or surface charge. Figure 7 presents the Anson plot of the *Q* vs. *t*^1/2^ and inset are the CC curves of total charge (*Q*) with respect to time (*t*) for the FTO, FMWCNTs, P3ABA, and FMWCNTs/P3ABA in 0.1 M KCl containing 5 mM K_3_[Fe(CN)_6_]. The EASA values were calculated from the slopes, yielding values of 0.2633 and 0.3120 cm^2^ for the FMWCNTs and P3ABA electrospun nanofiber films, respectively. These values are significantly higher than that of bare FTO (0.1768 cm^2^), indicating enhanced charge accumulation and improved electrochemical activity upon surface modification. The increased charge observed for the FMWCNTs nanofiber film can be attributed to the high electrical conductivity and large surface area of the carbon nanotube, which facilitate rapid electron transfer and provide additional active sites for the redox reaction of [Fe(CN)_6_]^3-/4-^ [63]. Similarly, the P3ABA nanofiber film exhibits a higher charge response than bare FTO, reflecting the intrinsic conductivity of the conducting polymer and its ability to promote interfacial electron transfer [64]. Notably, the FMWCNTs/P3ABA composite electrospun nanofiber film exhibits the highest charge accumulation among all investigated electrodes, demonstrating a pronounced synergistic effect between P3ABA and FMWCNTs. In this composite, the conducting polymer matrix ensures efficient contact with the electrolyte and favorable redox activity, while the embedded FMWCNTs act as highly conductive pathways that accelerate electron transport and reduce charge-transfer resistance [65,66]. Consequently, a steeper slope is observed in the Anson plot (*Q* vs. *t*^1/2^), corresponding to a substantially increased EASA. The EASA of the FMWCNTs/P3ABA composite reaches 0.4241 cm^2^, suggesting a greater number of accessible electroactive sites and improved diffusion of redox species at the electrode–electrolyte interface. These features are highly beneficial for electrochemical sensing applications, as they can lead to enhanced sensitivity, faster response, and improved analytical performance [67,68]. The chronocoulometric results confirm that the incorporation of FMWCNTs into the P3ABA nanofiber matrix effectively improves the electroactive sites, which led to enhance the electrochemical performance of the electrode.

### 3.3. Electrochemical Detection of Dopamine

**Figure 8:**
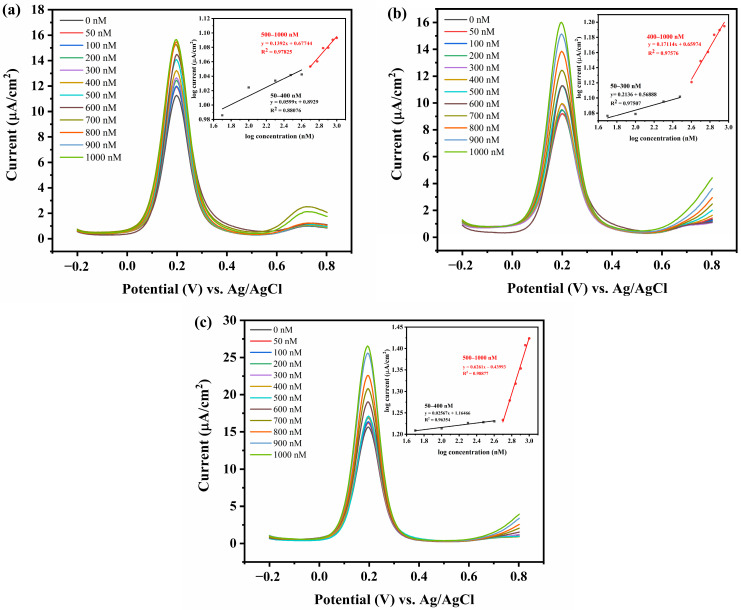
Differential pulse voltammograms of (**a**) FMWCNTs, (**b**) P3ABA, and (**c**) FMWCNTs/P3ABA electrospun nanofiber films upon successive addition with increasing concentration of dopamine in PBS solution containing 0.5 mM K_3_Fe(CN)_6_ and 0.1 M KCl ((insets) corresponding linear calibration curves of peak currents and DA concentrations).

The obtained electrospun FMWCNTs, P3ABA and FMWCNTs/P3ABA nanofiber films were used for electrochemical detection of dopamine at various concentrations of 50–1000 nM using the DPV technique in a potential range of −0.2 to 0.8 V at a scan rate of 5 mV/s in PBS containing 0.5 mM K_3_Fe(CN)_6_ and 0.1 M KCl. Figure 8 shows the DPV responses with linear plots of the response peak currents versus concentrations or calibration curves in the inset. The oxidation peak of the electrospun nanofiber films in a PBS solution containing 0.5 mM K_3_Fe(CN)_6_ and 0.1 M KCl was observed at about 0.25 V, which gradually enhanced upon addition of DA. Upon the addition of dopamine (DA), the oxidation peak of DA appeared at the same potential with an increased current density, indicating that FMWCNTs, P3ABA, and FMWCNTs/P3ABA composite play important roles in catalyzing the electrochemical oxidation of DA on the film surface [31]. The electrode modified with FMWCNTs exhibited a current response toward dopamine; however, the signal intensity was relatively low, suggesting limited electron transfer efficiency and a smaller electroactive surface area. Two linear ranges were observed at 50–400 nM and 500–1000 nM (Figure 8a), with corresponding sensitivities of 1.15 and 1.38 μA·cm^−2^·μM^−1^, respectively. The limit of detection (LOD) and limit of quantification (LOQ) were determined to be 2.04 μM and 6.79 μM, respectively. For dopamine detection using the conductive polymer P3ABA and the FMWCNTs/P3ABA composite, the enhancement in current response can be attributed to the interaction between the quinoid structure of P3ABA and oxidized dopamine species, such as dopaminechrome, which leads to an increased peak current upon DA addition [69,70]. The P3ABA film exhibited two linear ranges at 50–300 nM and 400–1000 nM (Figure 8b), with sensitivities of 1.64 and 1.48 μA·cm^−2^·μM^−1^, respectively. The LOD and LOQ were 1.77 μM and 5.89 μM, respectively. Notably, the P3ABA/f-CNT composite film (Figure 8c) showed sensitivities of 1.06 and 4.23 μA·cm^−2^·μM^−1^ in the linear ranges of 50–400 nM and 500–1000 nM, respectively, with LOD and LOQ values of 0.566 μM and 1.89 μM. These results indicate that the P3ABA/f-CNT composite exhibits higher sensitivity in the high concentration range and lower LOD and LOQ compared to the FMWCNT and P3ABA films. Therefore, the developed sensor was further applied for the detection of DA in human urine samples at various concentrations to evaluate its sensitivity and performance over a wider concentration range, particularly at lower concentrations. This is because dopamine levels in real human biological samples are typically present at relatively low concentrations.

### 3.4. Selectivity Study

**Figure 9:**
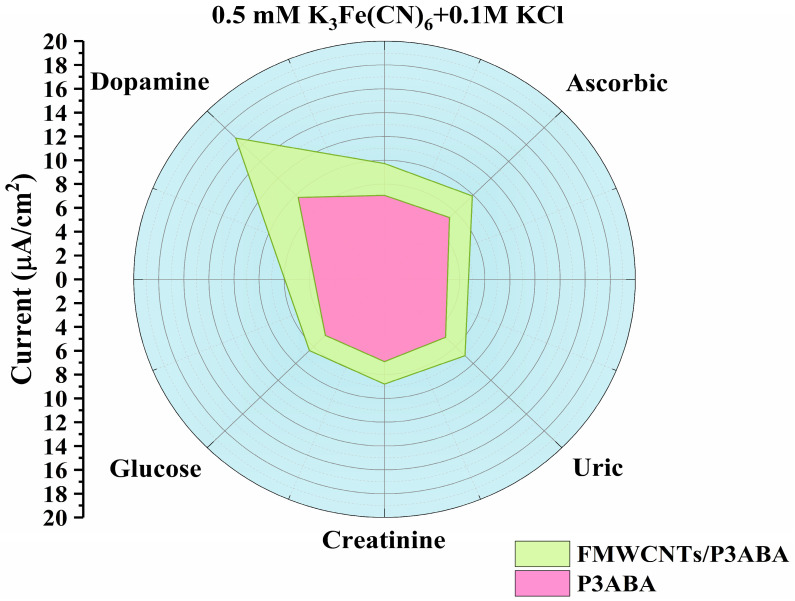
Selectivity histogram of P3ABA and FMWCNTs/P3ABA electrospun nanofiber films upon addition 1 mM of common interferents (AA, UA, Crn and Glu) in PBS solution containing 0.5 mM K_3_Fe(CN)_6_ and 0.1 M KCl.

The selectivity of the electrospun FMWCNTs/P3ABA nanofiber films was evaluated by the addition of common interfering agents, i.e., ascorbic acid (AA), uric acid (UA), creatinine (Crn) and glucose (Glu) in human urine. The DPV responses for the interfering study were presented in the electrochemical results noted in Figure S1. In addition, other interfering agents in the catecholamine group, such as tyramine, tyrosine, L-dopa, epinephrine (EP), and noradrenaline (NE), have similar molecular structures to dopamine (DA) that may also affect DA detection. However, due to the large size of these molecules and their low concentration in urine, their impact on DA detection in real samples may be considered negligible [10]. The common interferents in high concentration (1 mM) were used for detection. Figure 9 shows the selectivity histogram of the electrospun nanofiber film upon addition of all common interferents in PBS solution containing 0.1 M KCl and 0.5 mM K_3_Fe(CN)_6_ on the FTO substrate. The current responses after adding AA, UA, Glu, and Crn were not changed compared to the current response of the PBS solution containing 0.1 M KCl and 0.5 mM K_3_Fe(CN)_6_. However, after DA injection, the current response of the electrospun FMWCNTs/P3ABA composite nanofiber films significantly increased and were higher than that of the P3ABA films. These results indicate that the electrospun nanofiber of the FMWCNTs/P3ABA composite films exhibit good selectivity upon addition of common interfering agents.

### 3.5. Repeatability, Reproducibility and Stability of the Electrode

**Figure 10:**
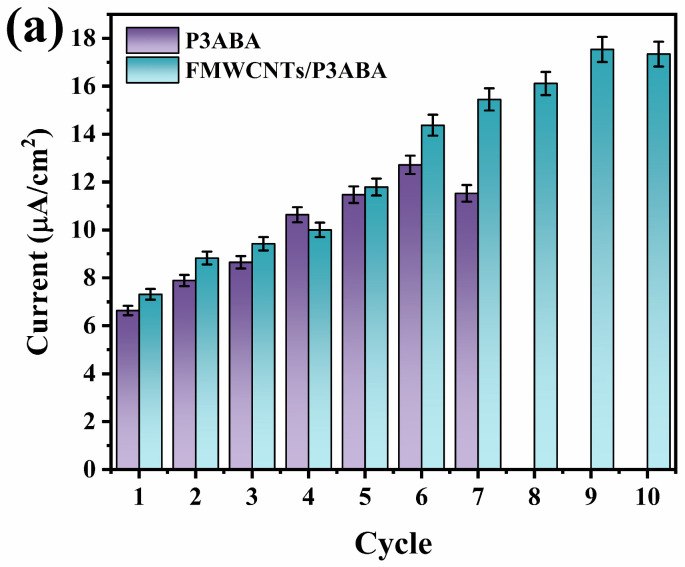

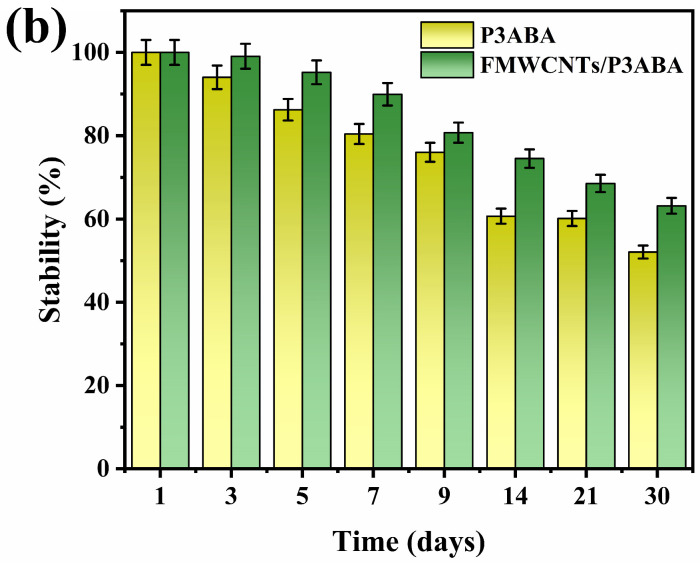
The representative histograms of (**a**) repeatability and (**b**) stability of the fabricated electrospun nanofibers of P3ABA and FMWCNTs/P3ABA composite films upon electrochemical detection of 0.1 mM DA.

The repeatability of the electrospun nanofiber films were evaluated by successive addition of 0.1 mM DA into the PBS solution containing 0.5 mM K_3_Fe(CN)_6_ and 0.1 M KCl for 10 cycles of DPV scanning as shown in Figure 10a for the P3ABA and FMWCNTs/P3ABA composite nanofiber film. The P3ABA presented repeatability up to 7 cycles, whereas the FMWCNTs/P3ABA presented repeatability up to 10 cycles. The improved repeatability of the FMWCNTs/P3ABA composite can be attributed to the incorporation of FMWCNTs, which enhanced the electrochemical consistence of the film.

In addition, the operational stability of the electrospun nanofiber films was evaluated by monitoring the current responses during the electrochemical detection of 0.1 mM DA in PBS containing 0.5 mM K_3_Fe(CN)_6_ and 0.1 M KCl. The measurements were carried out over a storage period of 30 days, as illustrated in Figure 10b. During this period, the prepared electrospun nanofiber films were stored at room temperature under dry conditions. The P3ABA and FMWCNTs/P3ABA films demonstrated the exact DPV responses, the current response of the P3ABA film was significantly decreased to below 80% after 7 days and below 70% after 9 days of storage. The current response of the FMWCNTs/P3ABA composite film remained at 80% for 9 days of storage. The stability improvement of the composite film could suggest the incorporation of the FMWCNTs with the P3ABA. Consequently, the fabricated electrospun FMWCNTs/P3ABA composite nanofiber film demonstrates promising electrochemical performance with acceptable stability, highlighting their potential applicability as an electrochemical dopamine sensor.

The reproducibility of the P3ABA and FMWCNTs/P3ABA nanofiber film was examined by measuring the DPV current responses of five similar fabricated electrodes in 10 nM DA in PBS solution containing 0.5 mM K_3_Fe(CN)_6_ and 0.1 M KCl. Low calculated relative standard deviation (RSD) values were obtained (4.05% for P3ABA and 1.21% for FMWCNTs/P3ABA). The FMWCNTs/P3ABA composite showed significantly improved reproducibility than that of the P3ABA film. The RSD value is acceptable which indicates good reproducibility of the electrospun FMWCNTs/P3ABA electrode.

### 3.6. Real Sample Analysis

**Figure 11:**
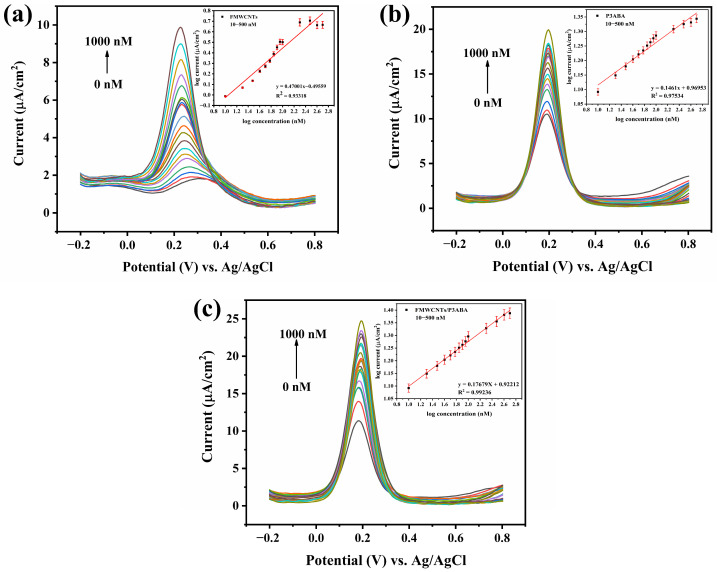
Differential pulse voltammograms of (**a**) FMWCNTs, (**b**) P3ABA and (**c**) FMWCNTs/P3ABA nanofiber films upon addition of various concentrations of DA in 5% *v*/*v* human urine in PBS solution containing 0.5 mM K_3_Fe(CN)_6_ and 0.1 M KCl. Insets are the corresponding linear calibration curves.

The fabricated electrospun FMWCNTs, P3ABA and FMWCNTs/P3ABA films deposited on FTO substrates were utilized for electrochemical dopamine (DA) sensing at various concentrations ranging from 10 to 1000 nM using the DPV technique. The measurements were carried out in a potential from −0.2 to 0.8 V versus Ag/AgCl with an effective scan rate of 5 mV s^−1^ in 5% (*v*/*v*) human urine in PBS containing 0.5 mM K_3_Fe(CN)_6_ and 0.1 M KCl. The corresponding DPV responses and calibration curves within the linear detection range are shown in Figure 11. A well-defined oxidation peak was observed at approximately 0.2 V versus Ag/AgCl, which is attributed to the electrochemical oxidation of dopamine [71,72]. The oxidation peak current increased systematically with increasing DA concentration, which demonstrated a clear concentration-dependent electrochemical response even in the presence of a complex biological matrix. The FMWCNTs/P3ABA electrode exhibited significantly higher oxidation peak currents than the pristine P3ABA electrode over the entire concentration range. This enhancement can be attributed to the synergistic effects between the conducting polymer and FMWCNTs, including improved electrical conductivity, enlarged electroactive surface area, and more efficient electron-transfer pathways [73,74,75]. In addition, the FMWCNTs could promote DA adsorption through π–π interactions between the aromatic rings of DA and the graphitic structure of CNTs, as well as electrostatic attraction, which collectively amplify the electrochemical signal [76]. The insets present the corresponding calibration plots of log current versus log DA concentration in the range of 10–500 nM. The FMWCNTs/P3ABA electrode demonstrated superior linearity with a correlation coefficient of R^2^ = 0.992, compared to R^2^ = 0.933 for the FMWCNTs, and R^2^ = 0.975 for the P3ABA electrode, indicating enhanced analytical reliability for DA determination in real biological samples. The apparent sensitivity, which calculated from the slope of the calibration curve, was determined to be 1.502 µA nM^−1^ cm^−2^ for the FMWCNTs/P3ABA electrode, which is considerably higher than that of the FMWCNTs and P3ABA electrode (1.114 and 1.340 µA nM^−1^ cm^−2^, respectively). The higher sensitivity reflects a stronger current response per unit concentration change and enables reliable detection of DA at low nanomolar levels.

Furthermore, the limit of detection (LOD) and limit of quantification (LOQ) were calculated based on the standard deviation of the blank signal (σ) and the slope (m) of the calibration curve, according to the equations of LOD = 3σ/m and LOQ = 10σ/m. These parameters represent the minimum concentrations of dopamine that can be reliably detected and quantitatively determined, respectively. The relatively steep slope and low background noise observed for the FMWCNTs/P3ABA electrode suggest significantly improved LOD and LOQ values of 1.753 and 5.1313 nM, respectively, compared with the FMWCNTs electrode (LOD = 2.519 nM and LOQ = 7.399 nM) and the pristine P3ABA electrode (LOD = 2.151 nM and LOQ = 6.519 nM). These results confirm the superior capability of the FMWCNTs/P3ABA composite electrode for trace-level dopamine detection. The enhanced analytical performance is primarily attributed to the doping effect of FMWCNTs, which promotes rapid electron transfer and effective signal amplification within the composite matrix [77,78].

**Scheme 1:**
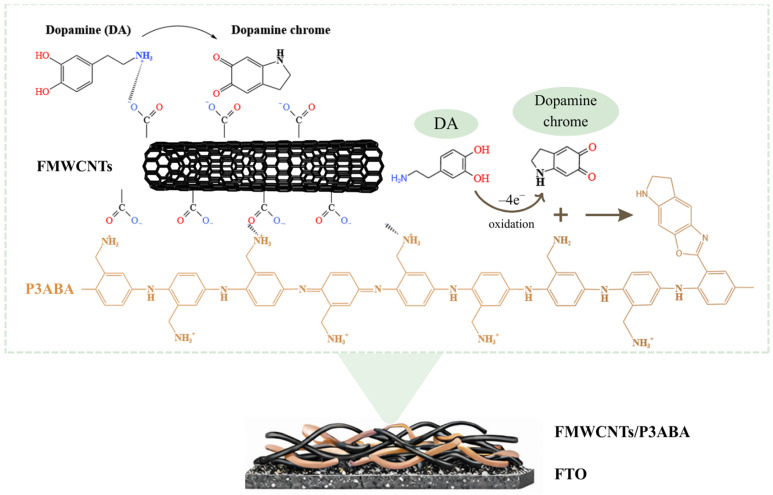
Proposed mechanism for detection of DA using FMWCNTs/P3ABA electrospun film.

The electrochemical oxidation of dopamine at the fabricated electrodes proceeds via a two-electron, two-proton process, which dopamine is initially oxidized to dopamine-o-quinone and subsequently transformed to dopaminechrome [79,80,81]. Compared with the pristine P3ABA electrode, the FMWCNTs/P3ABA electrode exhibited significantly enhanced oxidation peak currents. This enhancement can be attributed to the synergistic effect of FMWCNTs, which provide negatively charged carboxyl groups that promote dopamine adsorption through electrostatic attraction, along with π–π interactions among DA molecules, the conjugated P3ABA backbone, and the graphitic structure of FMWCNTs [82,83,84]. Furthermore, the electro-generated dopamine-o-quinone can interact with the amine groups of P3ABA through Schiff-base or Michael-type addition reactions, leading to the formation of dopaminechrome species stabilized at the electrode interface [85]. The proposed mechanism for detection of DA by the FMWCNTs/P3ABA composite electrospun film is illustrated in Scheme 1. The comparison of the fabricated FMWCNTs/P3ABA composite electrode with some previous reports of electrochemical DA sensor is presented in Table 2. It could be observed that the FMWCNTs/P3ABA presents higher sensitivity with lower LOD. Moreover, the good linear correlation obtained in human urine samples indicates that the electrochemical response of the FMWCNTs/P3ABA electrode is not significantly influenced by common interfering species in urine, such as UA and AA [86,87]. Overall, these results demonstrate that the FMWCNTs/P3ABA electrospun nanofiber film exhibits high sensitivity, low detection limits, good linearity, and stable electrochemically performance for DA detection in human urine, highlighting its strong potential for practical applications in real-sample analysis and non-invasive electrochemical sensing of neurotransmitters in the future.

**Table 2:** A comparison of electrochemical DA detection.

Electrode	Linear Range (µM)	Sensitivity (µA µM^−1^ cm^−2^)	LOD (µM)	References
PPy/MoO_3_	5–250	-	2.2	[8]
GO/SiO_2_@PANI	2–12	1.282	1.7	[21]
GCE/PEDOT/PANI	30–1000	-	4.58	[88]
GO/MWCNTs/PPy	0.05–70	0.52	0.29	[89]
PANI/CQDs	10–90	0.00802	0.1013	[90]
PA6/PAH/MWCNTs	1–70	0.158	0.15	[91]
GCE/PANI/AuNPs	20–100	-	16	[92]
FMWCNTs/P3ABA	0.01–0.5	1.502	0.001753	This work

Abbreviations: polypyrrole (PPy); molybdenum oxide (MoO_3_); graphene oxide (GO); silica oxide (SiO_2_); polyaniline (PANI); functionalized multi-walled carbon nanotubes (MWCNTs); glassy carbon electrode (GCE); poly(3,4-ethylenedioxythiophene) (PEDOT); carbon quantum dots (CQDs); polyamide 6 (PA6); poly(allylamine hydrochloride) (PAH); gold nanoparticles (AuNPs).

The reported physiological levels of DA in human urine are typically in the low nanomolar to sub-micromolar range. Clinical studies have shown that urinary DA concentrations in healthy individuals commonly range from approximately 20–300 nM, while the values can extend up to about 500 nM depending on age, diet, circadian rhythm, and metabolic conditions [93,94,95]. In certain pathological states or under pharmacological treatment, the urinary DA levels may increase up to 500 nM [96]. The electrospun FMWCNTs, P3ABA and FMWCNTs/P3ABA nanofiber films were employed as working electrodes for electrochemical detection of DA in human urine. The percentage recovery and RSD were obtained from the DPV peak currents of each concentration as shown in Table 3. The FMWCNTs/P3ABA composite electrode exhibited recovery values 98–108% with extremely low RSD (≤0.021%), demonstrating excellent accuracy and reproducibility in a complex biological matrix. In contrast, the FMWCNTs and the pristine P3ABA electrode showed lower recovery and higher variability, indicating more pronounced matrix effects. The previous reported DA sensors applied to human urine typically exhibited the recoveries of 83–101% with RSD values below 3% [97,98,99]. It could be observed that the FMWCNTs/P3ABA electrode shows comparable accuracy with significantly improved precision. These results highlight the effectiveness of incorporating FMWCNTs into electrospun P3ABA nanofibers.

## 4. Conclusions

The electrospun FMWCNTs/P3ABA composite nanofiber film fabricated on the FTO substrate demonstrated the great performance for selectivity and sensitivity electrochemical detection of DA in the human urine. Prior to use for DA sensing, the successful formation of the composite film and the effective incorporation of FMWCNTs were confirmed through TEM, BET surface area analysis, and zeta potential measurements. The electrocatalytic activity of the FMWCNTs/P3ABA was further studied by chronocoulometry. These results confirm the formation of the film and the existence of the FMWCNTs composite film. The electrochemical sensing performance evaluated by a DPV technique including selectivity, repeatability, reproducibility, stability and sensitivity of the composite film were improved due to the appearance of the FMWCNTs which could enhance the electron transfer and increase the electroactive surface area in the composite film. The FMWCNTs/P3ABA electrode exhibited a high sensitivity of 1.502 µA nM^−1^ cm^−2^ in the linear range of 10–500 nM (R^2^ = 0.992), along with a limit of detection (LOD) of 1.753 nM and a limit of quantity (LOQ) of 5.313 nM. Furthermore, the fabrication of FMWCNTs/P3ABA composite film exhibited great reliability in the detection of DA in human urine. Hence, the developed electrochemical DA sensor based on the FMWCNTs/P3ABA composite film showed the capability for dopamine detection in real biological samples.

## Figures and Tables

**Table 1:** Zeta potential values of FMWCNTs, P3ABA, and FMWCNTs/P3ABA electrospun nanofiber films in different electrolytes.

Zeta Potential (mV)		
	0.5 mM K_3_Fe(CN)_6_ + 0.1 M KCl	0.5 mM K_3_Fe(CN)_6_ + 0.1 M KCl + 20 nM DA
FMWCNTs	−2.4	−1.2
P3ABA	9.3	25.6
FMWCNTs/P3ABA	−45.7	−1.4

**Table 3:** Determination of DA in human urine (n = 5).

Electrode	Added Concentration (nM)	Measured Concentration (nM)	Recovery (%)	RSD (%)
FMWCNTs	10	8.72	93.92	0.053
	20	18.73	92.37	0.082
	50	47.69	96.10	0.095
	100	106.04	101.31	0.031
	200	195.98	95.77	0.089
	500	482.85	91.52	0.124
P3ABA	10	7.87	83.68	0.170
	20	16.32	86.44	0.112
	50	45.14	93.74	0.078
	100	108.10	101.69	0.058
	200	220.24	101.82	0.030
	500	498.08	99.94	0.020
FMWCNTs/P3ABA	10	9.86	99.39	0.021
	20	19.13	98.51	0.013
	50	49.17	99.57	0.006
	100	148.18	108.54	0.010
	200	198.20	99.83	0.006
	500	503.50	100.11	0.001

## Data Availability

The original contributions presented in the study are included in the article, further inquiries can be directed to the corresponding author.

## Supplementary Materials

The following supporting information can be downloaded at: https://www.mdpi.com/article/10.3390/bios16040226/s1. Figure S1: DPV curves for DA detection (1 mM) of the (a) P3ABA, and (b) FMWCNTs/P3ABA with the addition of the interferents (1 mM UA, AA, and Glu) in PBS containing 0.5 mM K_3_Fe(CN)_6_ and 0.1 M KCl.
