# Supplementary material for: Electrocatalytic Activity of Electrospun Multi-Walled Carbon Nanotubes/Poly(3-aminobenzylamine) Composite for Detection of Dopamine in Human Urine
Source: Biosensors (Basel). 2026 Apr 20;16(4):226. doi: 10.3390/bios16040226 (PMC13115534; doi:10.3390/bios16040226)
Supplement: Supplementary file 1 [file biosensors-16-00226-s001.zip › biosensors-4215175-supplementary.pdf]

Electrocatalytic Activity of Electrospun Multi-Walled Carbon Nanotubes/Poly(3-aminobenzylamine) Composite for Detection of Dopamine in Human Urine

Tharathip Khueanpech [1] and Saengrawee Sriwichai [1,2,*]

[1] Department of Chemistry, Faculty of Science, Chiang Mai University, Chiang Mai 50200, Thailand
[2] Center of Excellence for Innovation in Chemistry (PERCH-CIC), Faculty of Science, Chiang Mai University, Chiang Mai 50200, Thailand
* Correspondence: saengrawee.s@cmu.ac.th

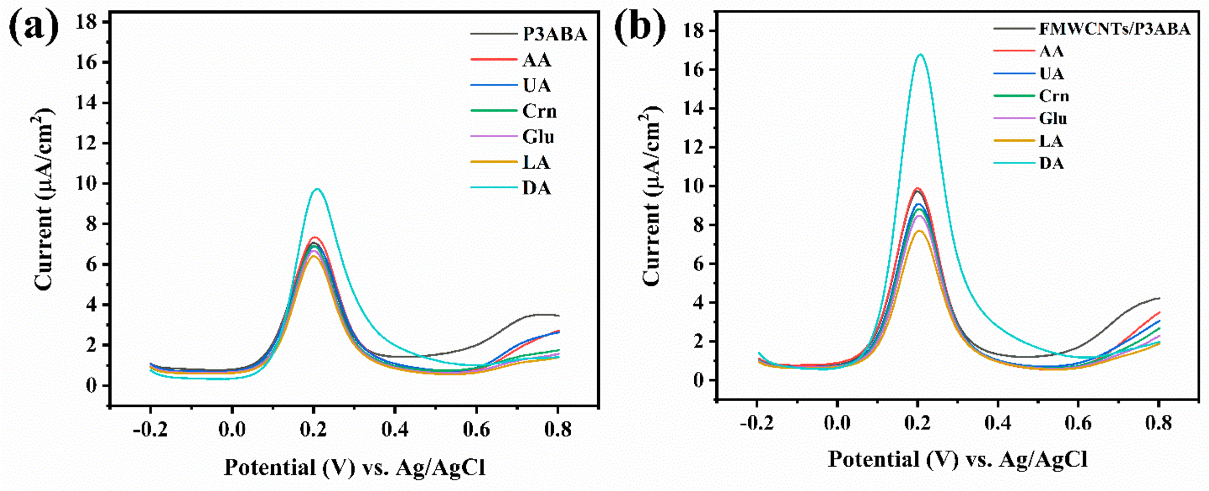

Figure S1. DPV curves for DA detection (1 mM) of the (a) P3ABA and (b) FMWCNTs/P3ABA with addition of the interferents (1 mM UA, AA, and Glu) in PBS containing 0.5 mM $K_3Fe(CN)_6$ and 0.1 M KCl.
